# Determinants of nutritional status of children aged 6–59 months in the case of Itang special woreda, Gambella, Ethiopia

**DOI:** 10.1038/s41598-024-59507-4

**Published:** 2024-05-07

**Authors:** Chekol Alemu, Habitamu Wudu, Meseret Abeje

**Affiliations:** 1 Department of Statistics, College of Natural and Computational Sciences, Gambella University, Gambella, Ethiopia; 2 Department of Biostatistics, Pharo Foundation, Assosa, Ethiopia

**Keywords:** Child malnutrition, Anthropometric index, Primary data, Multilevel ordinal logistic analysis, Diseases, Health occupations, Medical research, Risk factors

## Abstract

Nutritional status is one of the most important causes of improper physical and mental development in children. The study attempts to assess the factors affecting the severity status of children aged 6–59 months’ malnutrition based on the weight-for-age anthropometric index (z-score) and examine between-kebeles-level differences in determinants of the nutritional status of children. A community-based, cross-sectional study design was conducted from October 12 to November 12, 2022. A sample of 397 children aged 6–59 months primary data by applying multi–stage clustered sampling technique was used by considering their heterogeneity. The data were entered by SPSS and analyzed by using R version 3.4.0 and STATA 14.2 statistical software package using a multilevel ordinal logistic regression model and inferences were conducted at a 5% significance level. The results show that birth interval ≥ 24 months (OR = 1.431253, 95% CI 1.221337 1.6763421, P-value = 0.008), economic status of households medium (OR = 16.21466, 95% CI 1.221403 1.423929, P-value = 0.000), economic status of households rich (OR = 223.2856, 95% CI 1.34295 2.582325, P-value = 0.000), employment status of the mother unemployed (OR = 0.2291348, 95% CI 0.0529511 0.9966281, P-value = 0.049), No toilet facility (bush field) (OR = 0.3163329, 95% CI 0.1825356 0.5481975, P-value = 0.000), number of household members (OR = 0.9100682, 95% CI 0.8313481 0.9967315, P-value = 0.042), breastfeeding < 12 months (OR = 0.53803, 95% CI 0.322315 0.898135, P-value = 0.018), educational level of father Primary (OR = 4.601687, 95% CI 1.758009 2.22053, P-value = 0.000), educational level of father Secondary above (OR = 99.65229, 95% CI 2.533502 4.788896, P-value = 0.000) and geographical area (kebeles) were found to be important factors that affect a child's nutritional status between 6 and 59 months. 15% of the overall variation is attributable to the Kebeles level, according to two-level multilevel ordinal logistic regressions with estimates of the variation attributable to the Kebeles level equal to 0.569 and an intraclass correlation coefficient of 0.15. Due to the nature of the response variable random intercept model with random coefficients fitted the data adequately in predicting the severity status of children aged 6–59 months’ malnutrition for the multilevel ordinal logistic regression model analysis. So, the researcher recommended that implementing primary health care and nutrition programs that would fit each kebeles’ features in Itang Special Woreda to safeguard children from nutritional deficiency.

## Introduction

Nutritional status is the result of complex interactions between food consumption and the overall status of health and healthcare practices. Children's eating habits and women's and children's nutritional status are both influenced by a variety of socioeconomic and cultural factors^1^. The term malnutrition, generally, refers both to under nutrition and over nutrition but in this study the term is used to refer solely to a deficiency of nutrition ^2^.

Malnutrition has large costs in terms of missed GDP and increased spending, whereas boosting nutrition boosts productivity, and economic development, and reduces poverty by enhancing physical labor capacity, cognitive development, academic achievement, and health by lowering disease and mortality. A multi-sectorial development problem can lead to malnutrition and/or child death^3^. The different causes of malnutrition are interlinked and include immediate causes, underlying causes, and basic causes ^4^.

The regional summarized levels and trends of child malnutrition prevalence of 63 developing countries for the period of 1990s–2010s. They categorized these countries into five regions. Based on their order of underweight prevalence for the given period, the regions are presented as South Asia (61%), Sub-Saharan Africa (31%), East Asia (23%), Latin America and the Caribbean (12%), and, Near East and North Africa (11%) by using Bivariate and Trend analysis. The authors reported that except for Sub-Saharan Africa, there is some reduction in the level of malnutrition during the given period ^2^. As part of the welfare monitoring survey, the central statistical authority of Ethiopia is permanently providing data on the nutritional status of children every two years. 10,752 children under the age of five are eligible for height and weight measures, and the EDHS 2016 report states that 38% of these children are stunted (short for their age), 10% are wasted (thin for their height), and 24% are underweight (thin for their age). The high prevalence of malnutrition in Ethiopia points out the need to revisit the impression held by many people that malnutrition is not a problem in food-surplus areas. Development and implementation of preventive policies aimed at addressing child malnutrition should also consider food surplus areas of the country ^1^.

One of the most significant factors influencing health outcomes for people in any nation, including Ethiopia, is adequate nutrition^5^. However, malnutrition remains a big threat to almost all regions in Ethiopia particularly the Gambella region was one of the most highly affected by child stunting at 24% ^6^. These highest malnutrition rates in Gambella region in particular Itang special Woreda pose a significant obstacle to achieving better child health outcomes. Understanding the causes of malnutrition is necessary to reduce it. This study attempts to assess the nutritional status of children aged 6–59 months and to examine between-kebele level differences in determinants of the nutritional status of children aged 6–59 months and tries to check whether results agree with the findings of other scholars concerning the determinants of nutritional status in Itang Woreda using multilevel ordinal logistic regression model.

## Methods

### Study design and setting

A community-based cross-sectional study design was conducted from October 12 to November 12, 2022, at Itang special woreda, Gambella, Ethiopia.

### Data

The study population was all mothers/caregiver in the household, who has children aged 6–59 months, and who live in the study area for at least six months earlier to the time of data collection in the selected study site who give their consent were participated were included.

RUTF therapy should not be administered to children younger than six months old^7^. Height cut-offs are frequently used in SAM treatment programs to identify children likely to be aged less than 6 months and thus not eligible for treatment with RUTF. In cases, where there is more than one child aged 6–59 months in the same household, the youngest child was selected to avoid recall bias. Mothers/caregivers of children aged 6–59 months who have other health problems are critically ill, and those who did not live at least 6 months in the study area before the survey were excluded.

During the survey, the number of mothers/caregiver in the household, who had a children aged 6–59 months years living in Itang special woreda was 8685. Among the total children aged 6–59 months years residing in the 23 kebeles, 3652 children under five years living in the selected eleven sample kebeles were taken as the sampling frame. The study applies multi–stage sampling techniques. Initially, the 23 kebeles administrations of the district were clustered into two major sub-groups. The first cluster includes twelve kebeles that are found before the Baro River nearest to the town/main asphalt of Itang special woreda/ and the second cluster includes eleven kebeles that are found after the Baro River. From the twelve kebeles that are found before the Baro River, six kebeles (BILJAKOK, PILUAL, BAZIEL, DRONG, WAAR, and OKURA) were selected randomly for the study. Similarly, from the elven kebeles that are found after the Baro River, five kebeles (EBAGO, ADONG, ELIA, ALAHA, and ADIMA) were selected randomly taking into consideration their heterogeneity.

In both clusters, a total of eleven kebeles were included for the study chosen using simple random sampling, and the number of samples in each selected kebeles is determined using proportionate allocation.

Using ^8^ sample size determination formula, the total sample size (n) for the study was determined as $n=\frac{{\text{N}}}{1+{\text{N}}({e)}^{2}}=\frac{3652}{1+3652({0.05)}^{2}}=361$ Where, n = the sample size, N = the population size (N = 3652), e = sample size for precision (5% margin of error was used; considering the homogeneity of the study population).

Using this formula, 361 numbers of children below 5 years were determined from the Eleven Kebeles to fill out the questionnaire. However, fear of missing data/non-response rate, the sample size was determined to be 397, an increment of 10%. To this, ^9^ contend that it is wise to oversample 10–20% in the case when there is a non-response rate. Put differently, 10–20% could be added to the already calculated sample size to compensate for those that are unable to contact or not properly filled with^10^.

Finally, 397 numbers of children under the age of five were sampled for the structured interview from the eleven kebeles based on the sampling frames obtained from the Itang special woreda 2015 catchment population health offices (see Table 1).

**Table 1:** Statistical population and sample size of the study area.

No	Name of Kebeles	Numbers of children below 5 years	Sample size
1	EBAGO	114	12
2	ADONG	386	42
3	ELIA	229	25
4	ALAHA	117	13
5	ADIMA	87	10
6	BILJAKOK	414	45
7	PILUAL	556	60
8	BAZIEL	509	55
9	DRONG	547	60
10	WAAR	436	47
11	OKURA	257	28
Total		3652	397

Source: Itang special woreda 2015 catchment population health offices.

For stratum h (each year of a department), the number of samples is calculated by using proportional allocation. This leads to the following outcomes.$n_{hs} = \frac{{N_{hd} \times n}}{N}$ ^11^ and these samples in each selected kebeles are selected using simple random sampling.

Two types of instruments are used to collect the required data: a questionnaire developed by the researcher and anthropometric measurements including weight and age from healthy children aged 6–59 months during the study period. Portable weighing scales (Seca Model 881) are used to measure the weight of children. During the measurement, light clothing was worn, and weight was recorded to the nearest 0.1 kg^12^. Children who were unable to stand on the scale are weighed with the mother or caregiver, then the mother/caregiver was weighed alone, and the difference was used to obtain the net weight of the child. The heights of children are measured with an appropriate length scale with minimal clothes. The headpieces are brought down until they touched the head. Height and length are measured with trained health extension workers.

A variety of methods were commonly used for assessing the nutritional status of children aged 6–59 months such as anthropometric, clinical, dietary, and biochemical measurements but Anthropometric measurements (body dimensions and composition) are often used as proxies for assessing the eventual extent and severity of malnutrition. An outstanding general measure of a population's nutritional health is the weight-for-age anthropometric index. Moreover, weight-for-age was a composite index of weight-for-height and height-for-age ^13–15^. The dependent variable was the severity status of children aged 6–59 months' malnutrition based on a weight-for-age anthropometric index (z-score) categorized as severely undernourished (< − 3.0 Z-score), moderately undernourished (− 3.0 to − 2.01 Z-score) and Nourished (≥ − 2.00 Z-Score).

The socio-economic, demographic, maternal, and child health characteristics as the independent variables that influence the severity status of children aged 6–59 months of malnutrition were the mother's education, employment status of the mother, marital status of the mother, employment status of the father, economic status of the household, father's education, number of household members, area of residence, geographical area (kebele), sex of the child, age of the child, age of the mother at first birth, the birth interval of the child, birth type, vitamin a drop, during pregnancy given iron tablet/syrup, availability of toilet facility, source of water supply, had diarrhea in the two weeks before the survey and had a fever in the two weeks before Survey.

### Data analysis

Following data collection, data were checked and entered by SPSS and analyzed by using R version 3.4.0 and STATA 14.2 statistical software package. In this study the analyses focus on three outcomes of nutritional status for children aged 6–59 months; whether they are severely undernourished, moderately undernourished, or nourished. Two logistic regression models were performed separately. Firstly, the ordinal logistics regression model was employed to identify determinate factors of malnutrition among children aged 6–59 months and to predict the probability of children aged 6–59 months experiencing malnutrition. Finally, we assess the effect of determinant factors between kebele level differences on the prevalence of malnutrition using multilevel ordinal logistic regression model. All inferences were conducted at a 5% significance level.

### Ethical considerations

All procedures are carried out in compliance with the pertinent rules and journal standards. The ethical clearance approval letter was obtained from the Gambella University Institutional Review Board research directorate ethical approval committee (reference number: 258/GmU/2014 and due to date: 15/11/2014). The data collected from a structured questionnaire was developed by the researcher and anthropometric measurements including weight and age from healthy children aged 6–59 months during the study period health workers. The study was conducted with individual informed consent obtained from all subjects and their literate legal guardian. All methods were performed per the Declarations of Helsinki.

## Results

**Figure 1:**
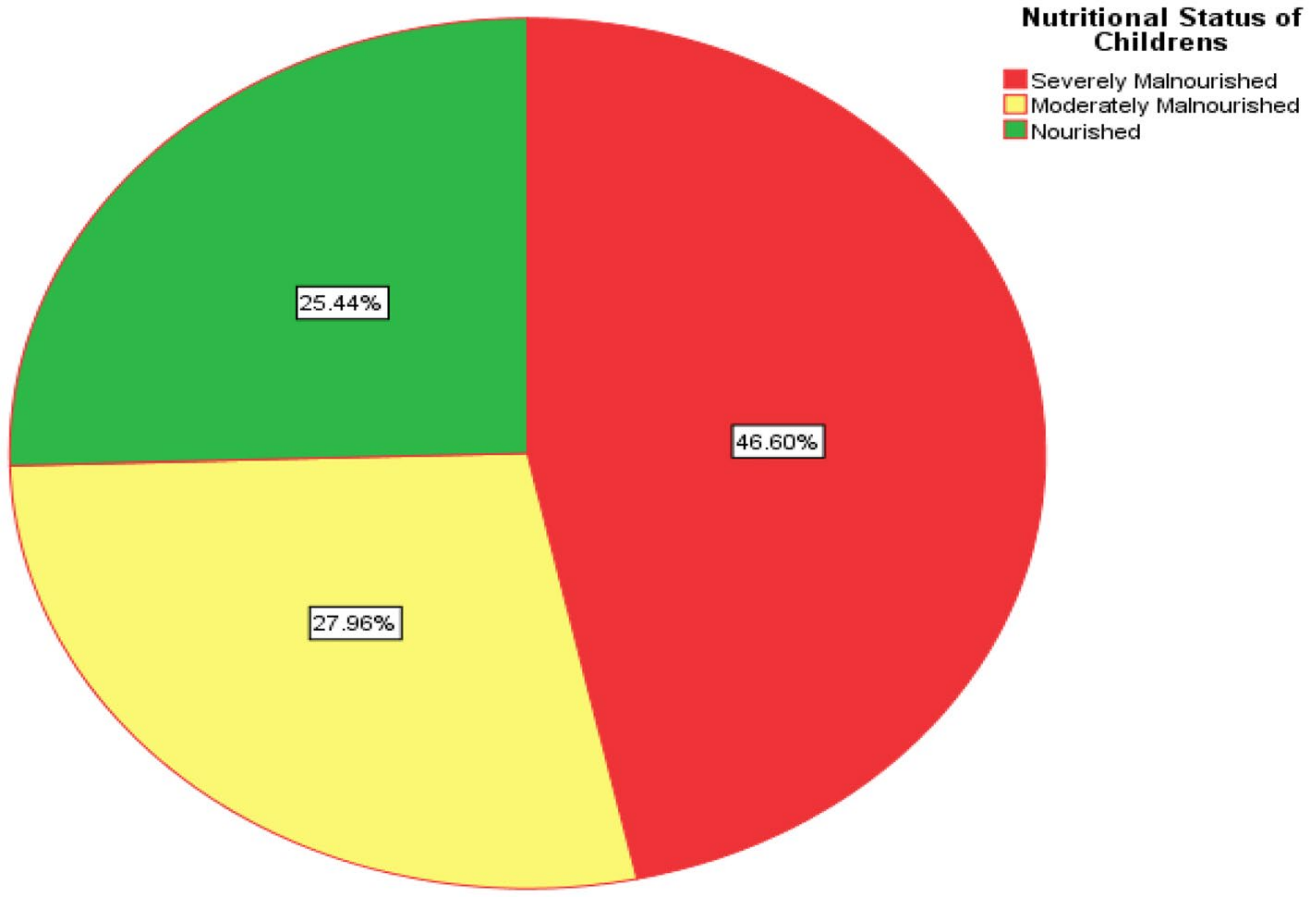
According to the WHO classification of nutritional status of children aged 6–59 months in Itang special woreda.

According to the WHO classification of children's nutritional status as shown in Fig. 1, the number of severely malnourished children aged 6–59 months is 185(46.60%) while 111(27.96%) are moderately malnourished and 101 (25.44%) are nourished.

### Results of cross-tabulation of data analysis

The total number of children covered in the study was 397. The sample encompasses both male and female children of which, 215 children (54.20%) were males and 182 children (45.80%) were females as shown Table 2.

**Table 2:** Results of descriptive analysis of socio-economic and demographic factors.

Covariates	Nutritional status			Total n (%)
	Severely malnourished n (%)	Moderately malnourished n(%)	Nourished n (%)	
Place of residence				
Rural	175 (50.2)	96 (27.6)	78 (22.2)	349 (87.93)
Urban	10 (20.6)	15 (30.4)	23 (49.0)	48 (12.07)
Mother’s education level				
No education	173 (51.8)	94 (28.1)	67 (20.1)	334 (84.13)
Primary	11 (24.7)	14 (30.9)	21 (44.4)	46 (11.48)
Secondary+	1 (5.9)	3 (17.6)	13 (76.5)	17 (4.28)
Sex of child				
Male	112 (51.9)	57 (26.5)	46 (21.6)	215 (54.20)
Female	73 (40.3)	54 (29.5)	55 (30.2)	182 (45.80)
Mother’s employment status				
Unemployed	184 (47.8)	110 (28.5)	92 (23.7)	386 (97.16)
Employed	1 (4.2)	1 (8.3)	9 (87.5)	11 (2.84)
During pregnancy, given iron tablets/syrup				
No	182 (48.6)	105 (27.9)	88 (23.5)	375 (94.84)
Yes	3 (13.0)	6 (28.3)	13 (58.7)	22 (5.44)
Economic status of the household				
Poor	144 (72.4)	50 (25.2)	5 (2.4)	199 (50.18)
Medium	35 (27.6)	44 (34.7)	47 (37.7)	126 (31.72)
Rich	6 (8.4)	17 (24.1)	49 (67.5)	72 (18.10)
Vitamin A supplement				
No	176 (49.2)	102 (28.4)	79 (22.4)	357 (89.94)
Yes	9 (23.5)	9 (23.5)	22 (53.0)	40 (10.06)
Father’s education level				
No education	172 (53.4)	92 (28.6)	58 (18.0)	322 (81.07)
Primary	11 (24.7)	12 (29.2)	19 (46.1)	42 (10.53)
Secondary+	2 (5.5)	7 (22.5)	24 (72.0)	33 (8.40)
Child’s age in month				
6–11	28 (65.1)	8 (18.6)	7 (16.5)	43 (10.77)
12–23	114 (56.5)	39 (19.6)	48 (23.9)	201 (50.65)
24+	43 (28.2)	64 (41.7)	46 (30.1)	153 (38.58)
Number of household members				
1–4	30 (35.3)	26 (30.6)	29 (34.1)	85 (21.42)
5–9	150 (52.0)	77 (26.9)	61 (21.0)	288 (72.54)
10+	5 (21.6)	8 (33.3)	11 (45.1)	24 (6.04)
Birth interval in month				
< 24	107 (61.5)	41 (23.6)	26 (14.9)	174 (43.83)
≥ 24	78 (34.8)	70 (31.4)	75 (33.8)	223 (56.17)
Age of the mother at the first birth				
< 18	136 (48.2)	80 (28.1)	67 (23.7)	283 (71.24)
≥ 18	49 (42.8)	31 (27.6)	34 (29.6)	114 (28.76)
Geographical area (kebele)				
EBAGO	6 (53.5)	4 (31.9)	2 (14.6)	12 (21.89)
ADONG	10 (24.6)	13 (30.3)	19 (45.1)	42 (14.44)
ELIA	14 (54.3)	6 (24.3)	5 (21.4)	25 (8.40)
ALAHA	3 (24.3)	4 (26.2)	6 (49.5)	13 (12.07)
ADIMA	6 (55.7)	2 (24.0)	2 (20.3)	10 (9.35)
BILJAKOK	25 (55.7)	11 (24.6)	9 (19.7)	45 (7.22)
PILUAL	33 (55.4)	17 (27.8)	10 (17.8)	60 (6.51)
BAZIEL	15 (27.3)	15 (27.3)	25 (44.5)	55 (5.21)
DRONG	32 (55.8)	19 (32.5)	9 (16.3)	60 (5.09)
WAAR	26 (55)	13 (27.5)	8 (17.5)	47 (4.85)
OKURA	15 (54.8)	7 (23.8)	6 (21.4)	28 (4.97)
Availability of toilet facility				
No facility	161 (57.0)	76 (27.0)	45 (16.0)	282 (71.01)
Have facility	24 (21.2)	35 (30.2)	56 (48.6)	115 (28.99)
Had diarrhea in the last two weeks before the survey				
No	157 (45.4)	96 (27.8)	92 (26.8)	345 (86.98)
Yes	28 (54.5)	15 (29.1)	9 (16.4)	52 (13.02)
Had fever in the last two weeks before the survey				
No	178 (46.8)	107 (28.1)	96 (25.1)	381 (96.09)
Yes	7 (42.4)	4 (24.4)	5 (33.3)	16 (3.91)
Source of water supply				
Unprotected	164 (56)	77 (26.3)	52 (17.8)	293 (73.73)
Protected	21 (19.8)	34 (32.4)	49 (47.8)	104 (26.27)
Father’s employment status				
Unemployed	183 (48.5)	108 (28.5)	86 (22.8)	377 (94.91)
Employed	2 (7.0)	3 (16.3)	15 (76.75)	20 (5.09)

As shown in Table 2 the proportion of children aged 6–59 months severely malnourished varied from one kebeles to another kebeles. The highest proportion (55.8%) of children aged 6–59 months severely malnourished were recorded in DRONG followed by ADIMA and BILJAKOK (55.7) and PILUAL (55.4%). However, the least proportion of children aged 6–59 months severely malnourished was observed in ALAHA (24.3%) followed by ADONG, (24.6%). The prevalence of children aged 6–59 months severely malnourished was different for different levels of mothers' education. About 51.8% of children aged 6–59 months severely malnourished were recorded for non-educated mothers while 24.7% of children aged 6–59 months severely malnourished before the age of 6–59 months were born from mothers with primary education. About 5.9% of severely malnourished children aged 6–59 months were observed from mothers that have completed secondary school and above. On the other hand, the highest proportion of children aged 6–59 months severely malnourished status (53.4%) were recorded from those who were uneducated fathers compared to 24.7% of children aged 6–59 months born to primarily educated fathers and 5.5% of children aged 6–59 months severely malnourished status were born from fathers who had completed secondary and higher educational level. The highest prevalence of severely malnourished children aged 6–59 months was observed as the educational status decreases, while the lowest prevalence of severely malnourished was recorded as the level of education increased in both cases of the mother's and husband's or father's educational level.

Based on Table 2 above, the experiences of children aged 6–59 months severely malnourished status were different based on the source of drinking water. A higher proportion of severely malnourished was recorded for children that drank unprotected sources of water and a relatively less prevalence of severely malnourished was observed for children aged 6–59 months that drank protected sources of water with a percentage of 56% and 19.8% respectively. The risk prevalence of severely malnourished was high (57%) for children aged 6–59 months who have no toilet facilities and the proportion of severely malnourished children aged 6–59 months was (21.2%) recorded as those having toilet facilities. The proportion (52.0%) of children aged 6–59 months severely malnourished was observed among households of family size 5–9 and the proportion of under-five child stunting was recorded from households of size 1–4 with a percentage of 35.3% and the prevalence of under-five child was observed among households of size 10 and above members which accounted for 21.6% of children aged 6–59 months were severely malnourished. The economic status of the household of parents showed different proportions of children aged 6–59 months severely malnourished exposure. Children aged 6–59 months born to poor families had the highest proportion of severely malnourished (72.4% for poor families and 27.6% for families with medium economic status) but children born to rich families had the least proportion of children aged 6–59 months severely malnourished (8.4% from rich families were recorded).

Table 2 also shows that the proportion of children found severely malnourished varies by the employment status of mothers. The higher proportion of the severely malnourished children aged 6–59 months were from unemployed mothers (47.8 percent). With regards to child age, the highest proportion of severely malnourished children aged 6–59 months were observed among those whose age group was between 12 and 23 months (56.5 percent) whereas the smallest percentage 28.2 percent of severely malnourished children which were observed among those whose age group is greater than 24 and above months.

The nesting structure is children between kebeles which resulted in a set of 11 kebeles with a total of 3652 children. The data used in this study consist of variables describing individuals as well as variables describing kebeles. Therefore, the statistical model used has to describe the data at both levels, to find the effect on nutritional status of both the individual children and the kebeles. Multilevel ordinal models were developed to analyse hierarchically ordered structured data. These models contain variables measured at different levels of the hierarchy. Random effects are used to model unobserved heterogeneity^16^. Children aged 6–59 months in this study were selected from different kebeles of Itang special woreda. Thus, there are two kinds of random variability in the data, variability between different children aged 6–59 months in a single Kebeles, and variability between different Kebeles.

### Random intercept model with random coefficients

We investigate whether level-one covariates have random or fixed effects across kebeles. The random coefficient model incorporates all of the variables included in the random intercept model^17^.

The results of the random slope estimates are given in Table 3 the Wald X^2^(24) = 211.83 with p-value = 0.000 indicates that at least one population parameter is significantly different from zero. Moreover, the likelihood ratio (LR) test shown at the foot of the table provides evidence that the logistic regression model with no intercept is rejected by the data. This means that the variance–covariance of random effects of the population is significantly different from zero. estimate of the fixed intercept is 0.569 and the log-odds of the probability of nutritional status when all level one covariates are zero in kebele j is given by 0.569 **+ **${\widehat{u}}_{j}$ where ${\widehat{u}}_{j}$ is a random intercept with a variance of 0.926 (indicated in the table as var(cons)) which is the between-kebeles variance and standard error 0 0.0000237. In the absence of level-one covariates, the status of each kebeles on nutritional status as compared to the average child's nutritional status measured with log odds depends on the sign of the random intercept ${\widehat{u}}_{j}$. When ${\widehat{u}}_{j}$ is positive the log odds of a child aged 6–59 months' nutritional status is higher than the average and when ${\widehat{u}}_{j}$ is negative the log odds of a child aged 6–59 months' nutritional status is less than the average. The individual kebele slopes of the economic status of the household vary with a variance of 0.256 as shown in Table 3.

**Table 3:** The random coefficients model *Wald X*^*2*^* (20)* = *211.83 Prob* > *X*^*2*^ = *0.0000.*

Nutritional status	Coefficients	Std. Err	Z	P > z	OR	[95% Conf. Interval]	
Place of residence							
Rural	− 0.3992752	0.3802747	− 1.05	0.294	0.6708061	0.3183512	1.413473
Main source of water supply							
Unprotected	0.4751253	0.3212323	1.48	0.139	1.608216	0.856862	3.018406
Toilet facility							
No facility (bush field)	− 1.15096	0.2805363	− 4.10	0.000*	0.3163329	0.1825356	0.5481975
Mother’s education level							
Primary	− 0.1787895	0.309041	− 0.58	0.563	0.836282	0.4563487	1.532525
Secondary+	0.4234788	0.5399556	0.78	0.433	1.527265	0.5300337	4.400737
Father’s Education level							
Primary	1.526423	0.3146905	3.75	0.000*	4.601687	1.758009	2.22053
Secondary+	4.601687	0.4137395	4.21	0.000*	99.65229	2.533502	4.788896
Age of the mother at the first birth							
≥ 18 years	0.0716353	0.1686158	0.42	0.671	1.074263	0.7719421	1.494985
Sex of the child							
Female	0.2077167	0.2840765	0.53	0.595	1.230864	0.8972057	1.688606
Mother’s employment status							
Un employed	− 1.473445	0.7474326	− 1.97	0.049*	0.2291348	0.0529511	0.9966281
Vitamin A supplement							
No	0.1508367	0.2840765	0.53	0.595	1.162807	0.3119339	1.688606
Number of household members	− 0.0942358	0.0463571	− 2.03	0.042*	0.9100682	0.8313481	0.9967315
Breastfeeding							
For < 12 months	− 0.6198347	0.2614231	− 2.37	0.018*	0.53803	0.322315	0.898135
During pregnancy given iron tablets/syrup							
No	0.1124242	0.5068763	0.22	0.824	1.118987	0.4143538	3.021895
Economic status of the household							
Medium	2.785916	0.289548	9.62	0.000*	16.21466	1.221403	1.423929
Rich	5.408452	0.4522622	11.96	0.000*	223.2856	1.34295	2.582325
Had fever in last two week							
No	− 0.37152553	0.4693839	− 0.79	0.429	.6896816	0.2748579	1.730569
Had diarrhea in last two weeks							
No	0.0677843	0.2468314	− 0.79	0.429	1.070134	0.2748579	1.73056
Birth interval							
≥ 24 months	0.3585501	0.08092056	4.431	0.008*	1.431253	1.221337	1.6763421
/cut 1 /cut 2	− 0.481241 1.995808	1.096739 1.12405	-0.44 1.81	0.661 0.070	− 0.481241 1.995808	0.0720202 0.8480074	1.950971 1.169785
var(_cons)	0.569002	0.000508	− 4.33	0.000*		0.8319534	0.8327818
Intra kebele correlation		0.087			0.038	2.34	0.010*

Random-effects parameters	Estimate	Std. Err	[95% Conf. Interval]				
RGN: Unstructured							
Var (Economic status)	0.256725	0.0000517	1.287331	1.287592			
var(constant)	0.9266952	0.0000237	2.524974	2.527321			
LR Chi^2^(10) = 66.22 p-value = 0.0000							

Model fit diagnostics							
N	Model	Df	LL (model)	Deviance	AIC	BIC	
397	Chi	23	− 563.9236	1127.8474	1177.84	1296.27	

‘*’ indicates statistical significance at a 5% significance level.

### Fitting multilevel proportional odds model

We need to fit a two-level multilevel proportional odds model to estimate the response variable nutritional status. In a two-level random intercept model we allow the cut points to vary at a different level of hierarchy. We take these random variations as a part of total random variation, i.e., we distribute the whole random variation into parts associated with different levels of hierarchies. In the present problem the estimation of nutritional status we use a two-level multilevel random intercept proportional odds model to find variation due division level of the child.

From the result shown in Table 4 multilevel model the estimation method MQL 1^st^ order produced can produce the biased estimate there, we used the PQL method for the estimation of the parameter of multilevel models. The value of Intra Class Correlation (ICC) of 0.15 show that 15% variation in the dependent variable "nutritional status" at the division level, (level two). By using the random intercept model with fixed predictors, the variation at the division level is 0.569. Multilevel ordinal logistic regressions were fitted to measure the nutritional status by using fixed effect and random effect. The fixed effect level 1, the model has fitted the results of level 1 (individual children level) were the same as the results of ordinal logistic regression. Next, we fit the two-level multilevel random intercept model for the dependent variable "nutritional status" and a set of fixed predictors. The odds ratios for the two-level random intercept model with fixed predictors are almost the same as for the level-one ordinal logistic regression model and are interpreted in the same manner. For the multilevel model the more important thing is the variation at a higher level, for our model the random variation at level two (kebele level) is Variance ${\tau }_{o}$ = 0.569 and Intra Class Correlation ICC** = **0.15. Intra-class correlation shows that 15% of variation lies at the division level. The level one residual has a standard logistic distribution with variance $\frac{{\pi }^{2}}{3}$ = 3.29. The much of total variation lies at level two units.

**Table 4:** Parameter estimates of the random intercept proportional odds with fixed predictors. *Wald X*^*2*^* (20)* = *921.83 Prob* > *X*^*2*^ = *0.0000.*

Nutritional status	Coefficients	Std. Err	Z	P > z	OR	[95% Conf. Interval]	
Place of residence							
Rural	− 0.0372981	0.1562598	− 0.24	0.811	0.9633889	0.9662273	1.30861
Mothers’ Education Level							
Primary	0.0677698	0.0765254	0.89	0.376	1.070119	0.4394756	1.243285
Secondary+	− 0.0836314	0.120795	− 0.69	0.489	0.9197702	0.7258692	1.165468
Father’s Education Level							
Primary	0.6316585	0.0785182	0.80	0.421	1.880727	0.9132694	1.24242
Secondary+	0.0575836	0.0968841	0.59	0.552	1.059274	0.8760731	1.025056
Mothers’ age at the first							
> 18	− 0.034893	0.0393425	− 0.87	0.383	0.9657087	0.8945817	1.043751
Sex of the child							
Female	− 0.0420281	0.0376729	− 1.12	0.265	0.9588428	0.890595	1.032321
Number of house h. members	0.015677	0.0078	2.02	0.043	1.015801	1.000389	1.0314494
Economic status of household							
Medium	− 0.5584807	0.0460666	− 12.12	0.000	0.5720776	0.5226885	0.6261334
Rich	− 1.306645	0.0585604	− 22.31	0.000	0.2707268	0.2413708	0.3036532
Breastfeeding							
For < 12 months	0.100011	0.0346642	2.88	0.007	1.105183	1.2301680	1.9928964
Availability of toilet facility							
No facility ((bush, field)	0.3062217	0.0709776	4.31	0.000	1.358283	1.181882	1.561013
Main Source of water supply							
Unprotected	0.0310264	0.1007232	0.30	0.76	1.031513	0.8459564	1.255508
Vitamin A Supplement							
No	− 0.0328332	0.0692191	− 0.47	0.635	0.9677	0.8449311	1.108307
Child’s mother gets iron syrup							
No	− 0.0376782	0.1149367	− 0.33	0.743	0.9630228	0.7687803	1.206343
Had diarrhea in last two week							
No	− 0.0254628	0.0595064	− 0.43	0.669	0.9748586	0.8675404	1.095453
Had fever in last two week							
No	0.0452537	0.1089729	0.42	0.678	1.046293	0.8675404	1.095453
Birth interval							
> 24	− 0.100011	0.0497864	− 2.01	0.022	0.904828	0.8207046	0.997573
_cons	2.289504	0.2462319	9.30	0.000	9.870041	1.806898	2.772109
Geography Var (-cons)	0.0155699	0.0087913				1.005162	1.048213
Var(e. dependent)	0.2849702	0.0139771				1.295441	1.368513

Var. Component	Variance (${\tau }_{o}$) = 0.569						
$\mathrm{ICC }= \frac{Var(Uoj)}{Var(Uoj)+\left(\frac{{\pi }^{2}}{3}\right)}=\frac{{\tau }_{o}}{{\tau }_{0}+3.29}=\frac{0.569}{0.569+3.29}$ ≈ 0.15							
LR test. linear model: Chibar2(01) = 22.17 prob ≥ chibar2 = 0.0000							

Model fit diagnostics							
N	Model	Df	LL(model)	Deviance	AIC	BIC	
397	chi	23	− 675.633	1351.266	1397.266	1506.216	

## Discussion

According to the study, there was a significant prevalence of severe malnutrition in rural areas of Itang Special Woreda among children between the ages of 6 and 59 months. The kebele of DRONG had the greatest percentage of severely malnourished children between the ages of 6–59 months (55.7%), followed by the kebeles of ADIMA and BILJAKOK (55.7%) and WAAR (55.0%) on the list. However, ALAHA kebele had the lowest percentage of severely malnourished children (24.3%) among children between the ages of 6–59 months, followed by ADONG kebele (24.6%). This discrepancy may result from the uneven distribution of infrastructure as well as the various socioeconomic and demographic factors that are present in individual kebeles. When compared to very malnourished children aged 6–59 months who consume protected sources of drinking water, children who drink unprotected sources of drinking water exhibit a more severe malnutrition level. However, compared to the reference group (who have toilet facilities), children who used toilets without any amenities were more likely to be seriously malnourished. Children whose moms were unemployed were far more likely to be exposed to severe malnutrition than children whose mothers were employed. Children whose mothers had only completed primary school or had no education at all were much more likely to suffer from severe malnutrition than children whose mothers had completed secondary school or more schooling. This finding seemed to be consistent with other studies ^2,18^. According to the study's research, children's improved nutritional health was favorably correlated with the caregiver's educational attainment. Higher education is probably to blame for this, as educated moms are more likely to be concerned about the health of their kids.; they tend to look after their children in a better way ^19^.

A study to determine the factors that contribute to child malnutrition in each developing region based on the experiences of 63 developing nations over 25 years. Women's education was one of the six criteria that were examined and found to be significant. They showed how the rise in female secondary school enrollment rates was thought to account for 43 percent of the overall 15.5% decline in child hunger rates in developing nations between 1970 and 1995 ^20^. Furthermore, the largest percentage of stunting was seen among dads with low educational attainment, whereas illiterate fathers had a far greater percentage of stunted children than fathers with secondary and higher educational attainment. For men with only a primary education, the percentage of children who are seriously malnourished is almost the same when it comes to less than three children. This fact might be connected to the fact that low income and an increase in the number of unemployed fathers make it difficult for fathers who are illiterate or have low levels of education to provide for their children's nutritional needs. Additionally, compared to children from wealthy households, under-five children from impoverished households are more likely to suffer from severe malnutrition. This result is in line with previous research^1^. Children who did not have a fever two weeks before to the survey date are far more likely to be seriously malnourished than those who did. This finding is consistent with other studies ^21^. In a similar vein, the study also revealed that children who did not have diarrhea for two weeks before the survey were noticeably more susceptible to severe malnutrition than children who did.

This study looked at kebele-level (individual and community) characteristics as important predictors of malnourished children aged 6–59 months. It demonstrates how crucial kebele differences are for malnourished children between the ages of 6 and 59 months. This study looked at regional differences in malnourished infants aged 6–59 months using a multilevel ordinal logistic regression method of analysis (kebele). The model indicates that there are differences in the status of malnourished children aged 6–59 months among kebeles; however, the main source of variation in the odds of having a malnourished child aged 6–59 months among different kebeles was found to be individual-level factors. These findings are consistent with most studies that have tried to differentiate contextual effects from compositional effects^22^ and support a major role for a community-level phenomenon as a strong influence on children aged 6–59 months malnourished.

## Conclusion

In predicting the severity status of children aged 6–59 months of malnutrition, due to the nature of the response variable random intercept model with random coefficients fitted the data adequately in predicting the severity status of children aged 6–59 months' malnutrition for the multilevel ordinal logistic regression model analysis. The economic status of the households, employment status of the mother, types of toilet facilities, breastfeeding, birth interval, number of the household members, educational level of the father, and geographical area (kebele) have a statistically significant effect on the status of malnutrition. Children aged 6–59 months' malnutrition remain a major public health/nutritional status problem in the rural part of Itang special woreda. Although there is a kebele disparity in children's health/nutritional status, it is observed that children living in rural parts of the country were at high risk of being malnourished. The result also suggested that children from poor families were more likely to be malnourished than children from rich families in Itang special woreda. Children younger than 11 months (infants) had better nutrition status than other age groups. This could be because of breastfeeding in the early stages of child growth. So, any intervention by governmental and non-governmental organizations that aims at improving the children aged 6–59 months nutritional status should consider Kebeles with high rates of children aged 6–59 months malnourished to avert under-coverage of the Kebeles deserve it. Moreover, further design and implement primary health care and nutrition programs which would fit the features of each kebele of in Itang special woreda to safeguard children from nutritional deficiency.

## Data Availability

The corresponding author will provide the datasets used and analyzed during the current research upon reasonable request.

## Abbreviations

DF: Degree of freedom
ICC: Intra class correlation
MQL: Marginal Quasi Likelihood
PQL: Penalized Quasi Likelihood
RUTF: Ready to use therapeutic food
SAM: Severe acute malnutrition
WHO: World Health Organization
